# Food supply and size class depending variations in phytodetritus intake in the benthic foraminifer *Ammonia tepida*

**DOI:** 10.1242/bio.030056

**Published:** 2018-03-14

**Authors:** Julia Wukovits, Patrick Bukenberger, Annekatrin Julie Enge, Maximillian Gerg, Wolfgang Wanek, Margarete Watzka, Petra Heinz

**Affiliations:** 1 Department of Palaeontology, University of Vienna, Althanstrasse 14, 1090 Vienna, Austria; 2 Department of Microbiology and Ecosystem Science, Terrestrial Ecosystem Research, University of Vienna, Althanstrasse 14, 1090 Vienna, Austria

**Keywords:** Carbon and nitrogen intake, Foraminifera, Laboratory feeding experiment, Size specific food intake, Stable isotope labeling

## Abstract

*Ammonia tepida* is a common and abundant benthic foraminifer in intertidal mudflats. Benthic foraminifera are primary consumers and detritivores and act as key players in sediment nutrient fluxes. In this study, laboratory feeding experiments using isotope-labeled phytodetritus were carried out with *A. tepida* collected at the German Wadden Sea, to investigate the response of *A. tepida* to varying food supply. Feeding mode (single pulse, constant feeding; different incubation temperatures) caused strong variations in cytoplasmic carbon and nitrogen cycling, suggesting generalistic adaptations to variations in food availability. To study the influence of intraspecific size to foraminiferal carbon and nitrogen cycling, three size fractions (125–250 µm, 250–355 µm, >355 µm) of *A. tepida* specimens were separated. Small individuals showed higher weight specific intake for phytodetritus, especially for phytodetrital nitrogen, highlighting that size distribution within foraminiferal populations is relevant to interpret foraminiferal carbon and nitrogen cycling. These results were used to extrapolate the data to natural populations of living *A. tepida* in sediment cores, demonstrating the impact of high abundances of small individuals on phytodetritus processing and nutrient cycling. It is estimated that at high abundances of individuals in the 125–250 µm size fraction, *Ammonia* populations can account for more than 11% of phytodetritus processing in intertidal benthic communities.

## Abstract (precis)

**Summary:** The food intake of *Ammonia tepida* (marine Protist) is strongly related to food supply and individual size. Specifically, juveniles play a major role in coastal phytodetritus processing.

## INTRODUCTION

Benthic foraminifera (eukaryotic single-celled organisms) have existed since the Paleozoic and colonize a variety of marine sediments, ranging from shallow water environments to the deep sea. Many species produce an inorganic shell (test), which is preserved in accumulating sediment layers after their death. Based on these attributes, benthic foraminifera serve as important tools for paleoenvironmental reconstructions, including estimations of organic matter fluxes in marine sediments and paleoproductivity studies (e.g. Altenbach, 1992; Herguera and Berger, 1991). They mainly feed on microalgae or phytodetritus (e.g. Bradshaw, 1961; Goldstein and Corliss, 1994; Lee et al., 1966) and their response to variations in phytodetritus fluxes can be observed on foraminiferal community compositions (Heinz and Hemleben, 2006) or metabolic activities (Graf and Linke, 1992; Linke, 1992). However, there is still a lack of studies quantifying foraminiferal carbon and nitrogen metabolism to improve the understanding of the connection between foraminiferal biomass and sediment organic matter fluxes.

High organic matter fluxes are characteristic for estuarine environments, where planktonic primary production can reach a gross particulate production of up to 176 g C m^−2^ year^−1^ (Tillmann et al., 2000). These fluxes show strong seasonal changes in quantity, characterized by high fluxes (500–2000 g C m^−2^ day^−1^) in summer months, typically ending with a high productivity peak in autumn, followed by a quick decrease towards winter months where they decline to zero. Microphytobenthos is additionally present throughout the year, with a strong reduction of abundant species in winter (Scholz and Liebezeit, 2012a). In such high productivity environments, foraminifera play an important role in sediment nutrient fluxes and phytodetrital carbon and nitrogen processing (Moodley et al., 2000; Wukovits et al., 2017). For example, Moodley et al. (2000) have evaluated that the genus *Ammonia* contributes with 1–7% to the processing of newly introduced, fresh phytodetrital carbon in an estuarine environment. Further, Cesbron et al. (2016) have determined that the foraminiferal aerobic carbon mineralization in intertidal mudflats can reach up to 7% of the total diffusive oxygen uptake.

*Ammonia tepida* is a common and dominant foraminiferal species in shallow water environments, showing seasonal oscillations in population densities and in size fractions (Alve and Murray, 2001; Murray and Alve, 2000). There is a high genetic variability within the *A.* *tepida* species-complex*,* and several molecular types have been previously identified (Hayward et al., 2004; Schweizer et al., 2008)*.* Morphological traits resemble those of molecular type T6 (Hayward et al., 2004).

*Ammonia* is an effective grazer of microalgae and its physiology, successful reproduction and survival is driven by factors including food availability, food quality or environmental temperature (Bradshaw, 1955, 1957, 1961; Lee et al., 1966). Laboratory feeding experiments can provide valuable information on their response to different food availability modes and were applied in this study to investigate phytodetritus intake of *A. tepida* under stable conditions. Specimens of *A. tepida* were fed in two different feeding modes (single, high quantity pulse versus constant feeding) and for the constant feeding at two different temperature cycles (18:20°C, 23:25°C, 12:12 h). Labelled food material (^13^C and ^15^N) was used and the cytoplasmic content of phytodetrital derived carbon (pC) and nitrogen (pN), as well as fluctuations in total cytoplasmic organic carbon (TOC) or nitrogen (TN), were analyzed in *A. tepida.* The aim was to investigate the response of this species to variations in phytodetritus flux (quantity or quality) and temperature, which provides fundamental information on the species-specific feeding ecology and fluxes of TOC and TN through foraminiferal communities. Additionally, phytodetritus intake of *A. tepida* individuals in different size fractions (125–250 µm, 250–355 µm, >355 µm) was compared. Smaller individuals of some other foraminiferal species take up more food particles than larger individuals and adult individuals show lower nutrient turnover rates (Langezaal et al., 2005; Nomaki et al., 2011). These intraspecific variations can be an important factor to understand carbon cycling on the seafloor and the role of foraminiferal nutrient cycling (Nomaki et al., 2006). To date there exist no data on phytodetritus-derived carbon and nitrogen intake across intraspecific size classes of foraminifera. Therefore, a second feeding experiment was carried out to observe pC and pN intake within three size classes of *A. tepida* (125–250 µm, 250–355 µm, >355 µm). For the comparison of the size-specific nutrient intake, pC and pN content were scaled with individual weight. Allometric scaling is widely used in theoretical biology to describe relationships of organismic mass or biovolume (X) with physiological parameters (Y), (here, pC and pN intake) according to the power function
(1)
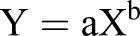
where ‘a’ is the scaling coefﬁcient and ‘b’ is the scaling exponent (log–log slope) (Kleiber, 1932; West et al., 1997; West and Brown, 2005). Here, a decrease of weight specific individual nutrient intake, due to higher nutrient demand and increased metabolic rates of smaller individuals was expected (b<1). Since *A. tepida* is a dominant protist in intertidal sediments, the impact of size class distributions on carbon and nitrogen cycling within *A. tepida* populations was estimated. This was done by extrapolating carbon and nitrogen data from the feeding experiment to size-specific abundances of *A. tepida* within sediment cores collected in spring 2015 at the Elbe Estuary. With this study, we wanted to extend the knowledge on patterns of phytodetritus intake and size-related pC and pN contents of the abundant foraminiferal species *A. tepida,* to obtain a more detailed understanding of the feeding ecology of intertidal foraminifera and their role in sediment carbon and nitrogen fluxes.

## RESULTS

### Experiment 1: Comparison of food uptake at a single high feeding pulse versus lower constant feeding

**Fig. 1.**
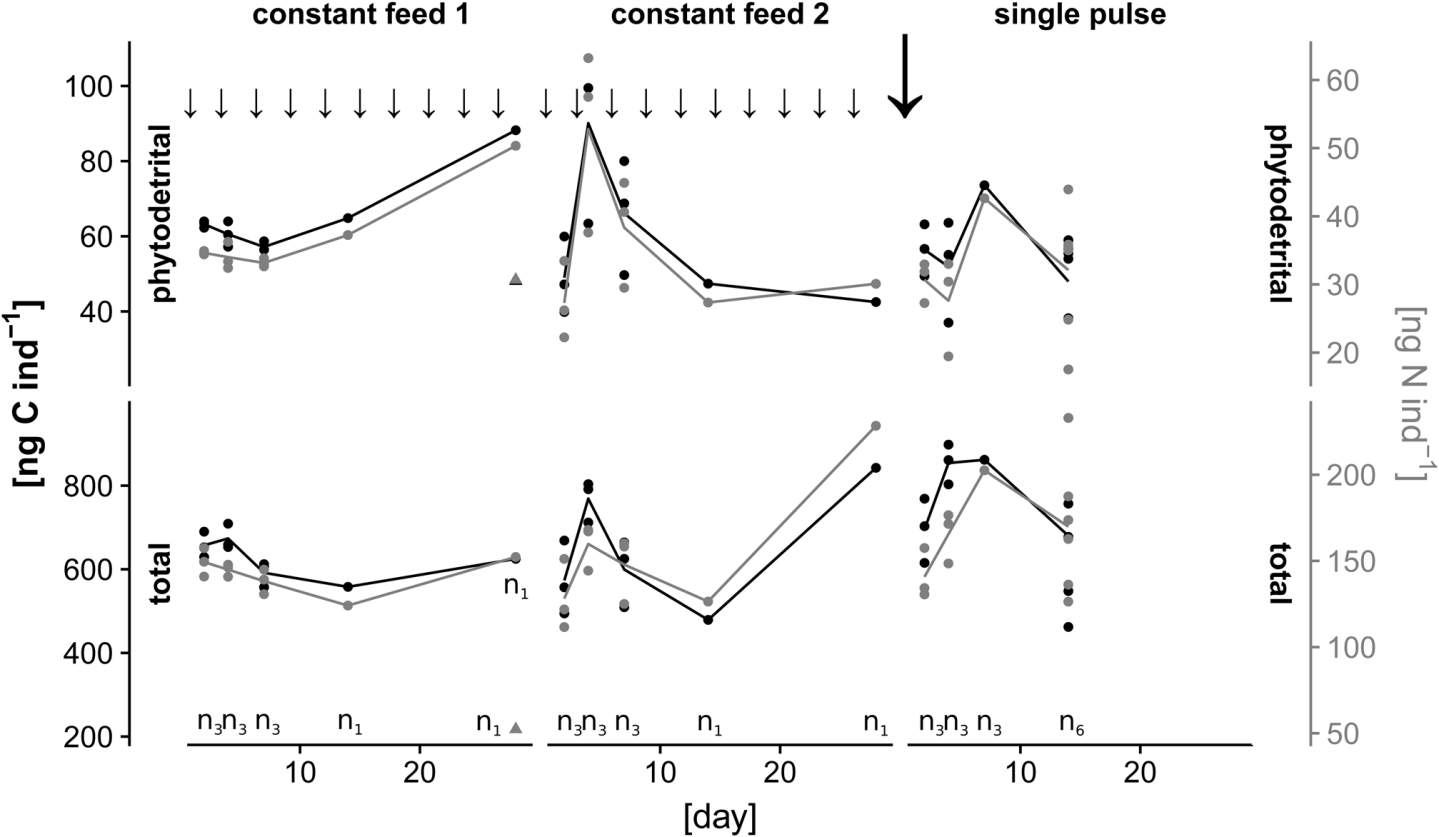
**Comparison of pC and pN (phytodetritus C and N intake) and cytoplasmic TOC and TN (total) in *A. tepida* for the different experimental approaches.** Arrows on the top indicate food supply, small arrows=2 mg, large arrow=6 mg phytodetritus dry weight. Black=carbon data, grey=nitrogen data, *n*=number of total replicates for the specific sampling point. Temperature settings were 20:18°C for constant feed 1; 25:23°C for constant feed 2 and 20:18°C for single pulse.

Culture conditions (O_2_, salinity, pH) were constant over time, but an increasing mortality of specimens was observed with time, so the initial total of 45 samples resulted in 37 elemental and isotope analyses. The individual values of pC or pN showed distinct patterns in the three approaches (Fig. 1). With constant feed settings, the lowest intake was observed at day 2 at constant feed 2 (25:23°C). At day 4 values for pC and pN peaked, followed by a steady decrease thereafter. For constant feed 1 (20:18°C), the highest pC and pN values were observed at the end, on day 28. For the single pulse approach (20:18°C), a peak for pC and pN appeared on day 7. Cytoplasmic TOC and TN content of the analyzed specimen also showed some variations over the course of the treatments. Noticeable is the increase in TOC and TN at constant feed 2 (25:23°C) in comparison with the stable patterns at constant feed 1 (20:18°C) and the generally highest TOC and TN values for the single pulse approach at day 4 and 7.

In one of the replicates on day 28 in constant feed 1 (20:18°C), the number of *A. tepida* individuals increased from the initial 55 specimens to 88 individuals, including 35 individuals of small test size with intense green cytoplasmic coloration, determined as young individuals. Those 35 young individuals were pooled together in an individual sample (Fig. 1, triangles).

**Fig. 2.**
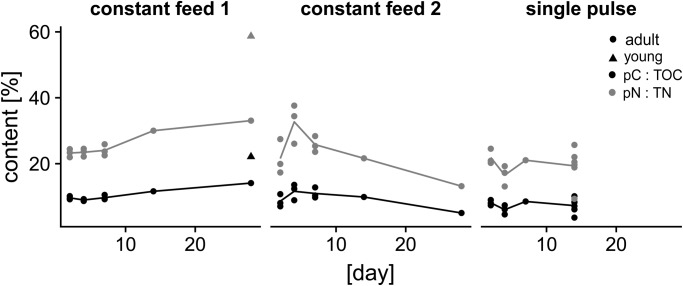
**Relative amounts of pC:TOC and pN:TN in percent for Experiment 1.** Constant feed 1 (20:18°C) day 2–7, *n*=3; day 14 and 28, *n*=1, constant feed 2 (25:23°C): day 2–7, *n*=3; day 14 and 28, *n*=1, single pulse (20:18°C): day 2–7, *n*=3; day 14, *n*=6).

**Fig. 3.**
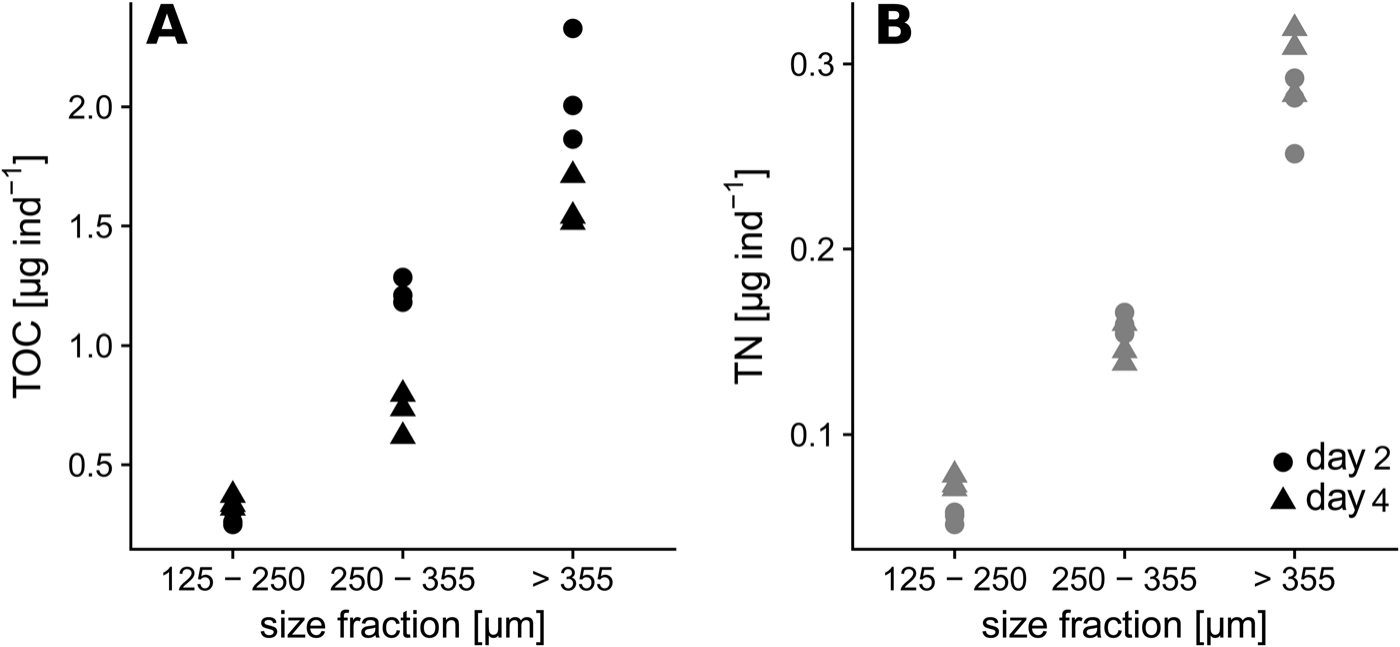
**Individual content of foraminiferal cytoplasmic organic carbon (TOC) and nitrogen (TN) per size fraction analyzed in Experiment 2.** Circles show data for day 2 (*n*=3 for each size fraction and day), triangles show data for day 4 (*n*=3 for each size fraction and day).

The maximum diameter of young individuals was 243 µm (±23) and for adults 375 µm (±72). The fractions of pC:TOC and specially pN:TN (percent), were higher in young individuals than in adults (Fig. 2). Generally, the fraction of pN:TN was considerably higher than pC:TOC in all samples (Fig. 3).

### Experiment 2: Size specific cytoplasmic TOC and TN and phytodetritus intake

Size class specific contents of cytoplasmic TOC and TN were strongly increasing with size class, but did not show significant differences between day 2 and day 4. Values for TOC and TN data (day 2 and day 4) of Experiment 2 per size fraction are shown in Fig. 3. Individual phytodetritus intake as pC and pN per size fraction showed slight but not significant deviations between days (Table 1). Distributions of living *A. tepida* specimen as counted from sediment cores collected parallel with specimen for the feeding Experiment 2 showed highest numbers in the 125–250 µm size at all three stations (Table 1). Stations 1 and 2 were characterized by high densities of living *A. tepida*, while Station 3 showed very low total abundances. Due to these very low numbers of individuals per sediment volume, the ratios of foraminiferal size fractions might be inaccurate for the core from Station 3. Station 3 was therefore excluded from extrapolations of carbon and nitrogen budgets. Extrapolated values to total abundances of living *A. tepida* individuals resulted in total population TOC ∼50 µg cm^−3^, pC ∼6.20 µg cm^−3^, TN ∼8.8 µg cm^−3^ and pN ∼1.10 µg cm^−3^ (Station 1) and TOC ∼55 µg cm^−3^, pC ∼7.90 µg cm^−3^, TN 10 µg cm^−3^ and pN ∼1.50 µg cm^−3^ (Station 2).

**Table 1.**
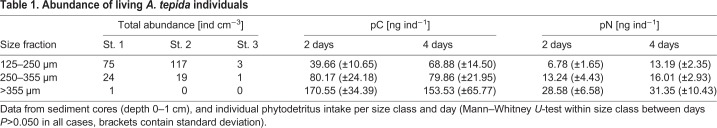
**Abundance of living *A. tepida* individuals**

**Fig. 4.**
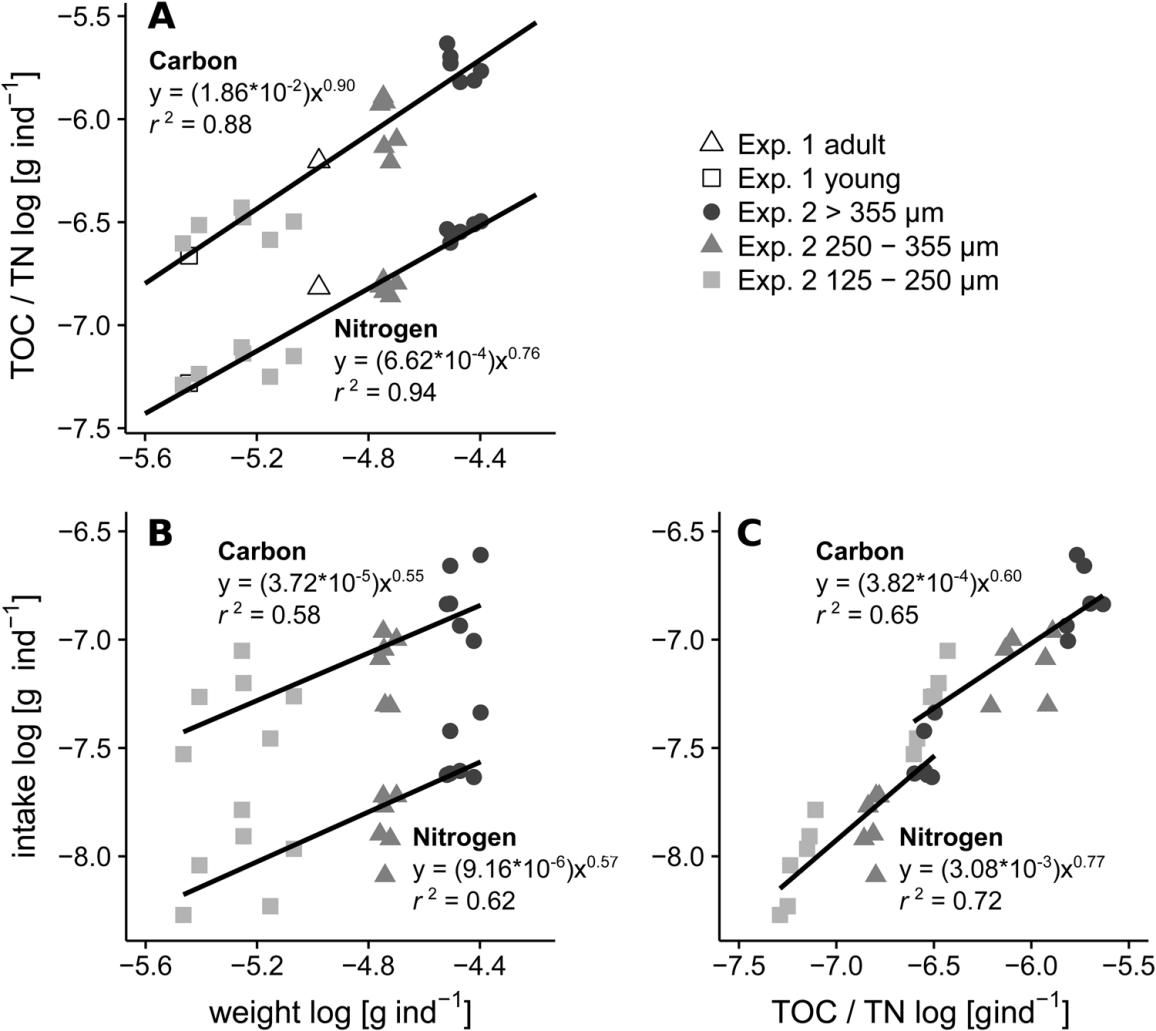
**Size-related general least squares (GLS) regressions.** (A) Individual TOC and TN content per individual dry weight, from Experiment 1 (constant feed 1 at 20:18°C, day 28) and Experiment 2 (day 2 and day 4, *n*=3 per sampling day). (B) Carbon and nitrogen uptake rates per individual dry weight and (C) individual carbon and nitrogen intake per individual TOC and TN content. Equations show log back transformed scaling coefficients. All regressions were significant (*P*<0.01). Ferret diameter of adult individuals Exp. 1=375 µm (±72) and young individuals=243 µm (±23). Exp. 2 size ranges are mesh widths of sieves.

Assumptions for the application of general least squares (GLS) regressions for specific weight, TOC, TN, pC and pN were met. Weight-specific TOC content of *A. tepida* was relatively similar across size fractions (Fig. 4A, Carbon, b ∼0.90), while the weight-specific TN content decreased with increasing size with a proportionality of ∼0.75 (Fig. 4A, Nitrogen). Phytodetritus intake scaled similar for pC and pN per individual dry weight (proportionality between 0.55 and 0.57), but the weight-specific intake became relatively lower with increasing foraminiferal weight (Fig. 4B). Interestingly, the TN related pN uptake also showed a ∼0.75 proportionality (Fig. 4C, Nitrogen).

**Fig. 5.**
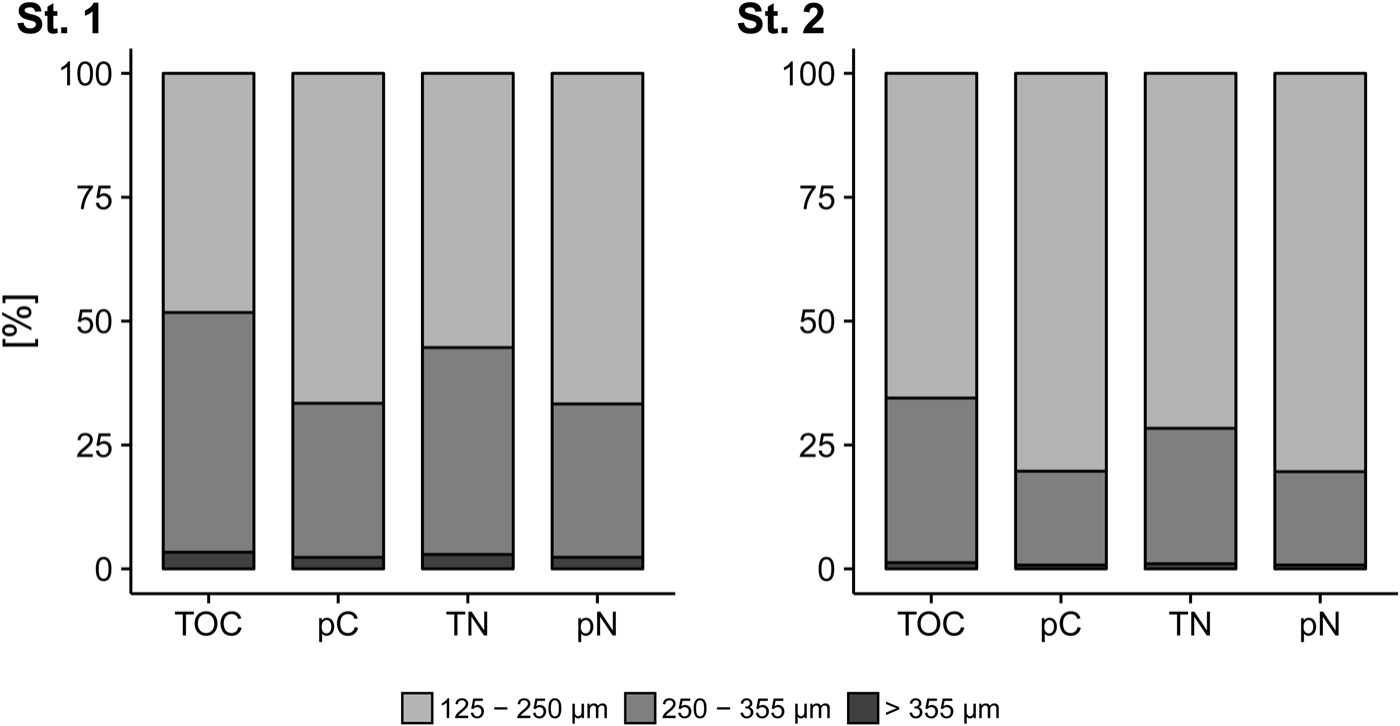
**Ratios of carbon and nitrogen pools in populations of *A. tepida***. Relative contributions of size fractions of *A. tepida* to total population TOC and TN pools and phytodetritus intake (pC, pN) at Station 1 and Station 2, as extrapolated from feeding Experiment 2 to relative abundance data from stained sediment cores.

Extrapolations of individual TOC, TN, pC and pN to natural populations of *A. tepida* of Station 1 and Station 2 (Station 3 was excluded due to low *A. tepida* abundances) show that the gross TOC and TN pool and phytodetritus processing of the *A. tepida* community in April 2015 was dominated by the smallest size fraction (125–250 µm) at both stations with a specifically high pN intake at Station 2 (Fig. 5).

The raw isotope and elemental data for both experiments can be found in Table S1.

## DISCUSSION

### Response to different feeding modes in *A. tepida*

Generally, empirical studies show that foraminiferal feeding behavior and phytodetritus intake varies between species according to the primary productivity of the habitat or their adaptations (Moodley et al., 2002; Witte et al., 2003; Enge et al., 2011, 2014, 2016). Different strategies include the rapid response to pulses of food supply by high food uptake, or constant feeding at moderate food concentrations (Altenbach, 1992; Linke, 1992). The results of this study clearly show different metabolic responses of *A. tepida* to different feeding modes and temperatures as expressed in distinct patterns of cytoplasmic pC, pN, TOC and TN levels (Fig. 1). Constant food supply at 20°C caused a prolonged uptake of phytodetritus at stable levels of TOC and TN throughout the experiment, indicating that the costs for metabolic maintenance were covered. But there was no increase in individual biomass over the course of time in this approach, therefore individual growth was not highly supported by the constant supply of *D. tertiolecta* detritus, at least at the adult stage. On the other hand, the single, high pulse of phytodetritus caused an increase in individual biomass, particularly in TOC (Fig. 1). Differences in pC and pN between constant feed 1 (20:18°C) and the single pulse suggest different rates of food turnover or usage of cellular storage products. The decrease of TOC and TN at the end of the single pulse approach is most likely caused by exhaustion of such storage products and limited subsequent uptake of the aging and degrading food particles. A recent study with *A. tepida* fed with frozen diatoms also showed a comparable pattern of cytoplasmic carbon increase following a single food pulse of 587 mg C m^−2^ (LeKieffre et al., 2017). In that study, the individual carbon content showed a stronger increase than we found in the present study, which was also followed by a decrease in TOC after the seventh day of incubation. In contrast to the present study, the food derived ^13^C signal did not decrease simultaneously with the cytoplasmic carbon content after day seven. This is most likely related to the different breakdown dynamics of soft chlorophyte cells (the food source in this study) in contrast to the diatoms with their surrounding frustule used by LeKieffre et al. (2017) and the conceivably slower turnover of diatom derived ^13^C label.

A similar response – a strong increase in individual carbon following a high pulse of food (1.1 g C m^−2^) with subsequent loss of cytoplasmic carbon – was shown earlier in *Cribrostomoides subglobosum* from the Norwegian Sea (Altenbach, 1992). Here, the individual organic carbon in *C. subglobosum* almost doubled within 2 days after food supply. This was interpreted as a quick response to the food pulse, resulting from the opportunistic feeding strategy of *C. subglobosum*, coherent with the ability of some foraminiferal species to take advantage of high food quantities and build up reserves as a response to fluctuating food availability (Altenbach, 1992; Gooday et al., 1990; Heeger, 1990; Hickman and Lipps, 1983; Lee et al., 1966).

Opportunistic foraminiferal species have been further identified based on their strong response to pulses of live algae, which triggered reproduction, or increased population densities in laboratory feeding experiments (Heinz et al., 2001). These observations were consistent with findings on population fluctuations in nature, where the increase in population densities coincided with phytoplankton blooms in these species (Heinz and Hemleben, 2006; Kitazato et al., 2000). Considering the large test size of the young *A. tepida* individuals (∼243 µm) in this study, on the last day of constant feed 1 at 20:18°C (Experiment 1), the reproduction most likely occurred randomly at the beginning of the incubation period. Laboratory grown juvenile specimens of *A. tepida* showed a test size of ∼125 µm (8 chamber stage) about 2 weeks after release from the parental individual (Goldstein and Moodley, 1993). However, the amount of newly released juveniles (35) indicates that more than one adult individual must have reproduced, since it was described that asexual reproduction results in the release of a maximum 12–24 calcified juveniles (Stouff et al., 1999).

*A. tepida* is also considered as an opportunistic feeder with a strong response to food input (Lee et al., 1966). Our study revealed a pattern of phytodetrital nutrient uptake consistent with such a strategy in the single pulse approach, with increased food uptake and individual biomass until day 7. In contrast, the constant input of lower amounts of food resulted in reduced metabolic activity (reduced gain of biomass, steady and low food intake compared with the high single pulse, Fig. 1). Regulation of metabolic activity in *A.* *tepida* to survive under unfavorable environmental conditions has been reported recently (Lekieffre et al., 2017). Here, a lowering of metabolic activity to a minimum might have been induced by unfavorable nutrient availability. Typically, strategies including reduced metabolic activity followed by strong responses to high fluxes of food are observed in the deep sea, where food is a limiting factor (Graf and Linke, 1992; Linke, 1992). The intertidal is not considered nutrient limited and the results of this experiment show a flexibility in the response of *A. tepida* to different feeding modes.

Other environmental factors, like temperature, can also have a strong effect on the rates of carbon and nitrogen intake and metabolism in *A. tepida*, as demonstrated by the strong variation of pC and pN patterns at constant feed 1 (20:18°C) and constant feed 2 (25:23°C) at the same levels of food supply (Fig. 1). This can be explained by the strong effect of temperature on metabolic activity, specifically on respiration rates controlling foraminiferal carbon processing. Thermal response curves revealed a steep rise of oxygen consumption in *A. tepida* between 20°C and 30°C (Bradshaw, 1961) and in respiration of *A. tepida* which was substantially higher at 23°C compared to 7°C (Cesbron et al., 2016). This effect of temperature has also been observed on the level of carbon processing in *A. tepida* in a former feeding experiment (Wukovits et al., 2017). There, a single low food pulse (∼220 µg C m^−2^) at three different temperatures (20°C, 25°C, 30°C) resulted in the highest levels of pC in *A. tepida* at 25°C. In the current study, the constant food supply caused a similar rapid rise in pC at 25°C, but only until the fourth day, and it dropped on the following days despite constant food supply. The former study was carried out in vials containing solely phytodetritus (Wukovits et al., 2017), while in this study a heat sterilized sediment layer was present. Sediment composition reportedly influences locomotion speed in *A. tepida* (Jauffrais et al., 2016), while live prey and inorganic matter slow foraminiferal movement and high organic matter content increases locomotion speed. Therefore, the high content of inorganic particles in the sediment in Experiment 1 (in contrast to exclusively organic matter) probably decreased foraminiferal food particle accumulation by decreasing locomotion through the sediment. However, the effect of increased temperature is evident in both studies. Increased environmental temperatures might therefore alter the organic matter processing by *A. tepida* in its natural environment and cause shifts in intertidal food webs. The rapid decrease in pC and pN in constant feed 2 (25:23°C) in this study might also be explained by bacterial presence in the experimental setup. The test (calcified shell) of the species *Ammonia* is characterized by immersions surrounding the umbilical plug which are often filled with organic material and might therefore host associated bacteria. Despite careful cleaning of the specimens, bacteria can remain in these cavities and reproduce during the course of the experiment, especially at high temperatures. These bacteria can contribute to the mineralization of organic matter, or the phytodetritus in the experiment, and get isotopically enriched. Bacteria can also serve as a food source for *Ammonia* species (Brouwer et al., 2016; Langezaal et al., 2005; Pascal et al., 2008) and the ingestion of lower isotope enriched bacteria can cause a dilution of the isotope signal or lower the pC and pN content in the foraminifera.

Altogether, this experiment showed clear adaptations of *A. tepida* to variations in food availability at the level of phytodetritus intake and internal carbon and nitrogen reserves of adult individuals. This implies a generalistic adaptation of *A. tepida* to different levels of food supply, with a strong sensitivity of food intake and processing to environmental temperature.

The mortality of the individuals in Experiment 1 was relatively high (see Fig. S1), compared to other laboratory experiments with *Ammonia tepida* (Geslin et al., 2014; LeKieffre et al., 2017; Nardelli et al., 2014). The culture conditions in Experiment 1 of this study were set up as close as possible to natural conditions and followed the literature documenting successful cultivation of intertidal foraminifera (Boltovskoy and Wright, 1976; Lee et al., 1961, 1966; Muller and Lee, 1969). These suggest cultivation in filtered habitat seawater, to ensure the supply of micronutrients and specific associated bacteria, which are necessary for growth and reproduction. It is known that ecological strategies involving the production of juveniles in large quantities implicate a high mortality rate during ontogenesis, caused by environmental factors and genetic variability within the population (Demetrius, 1978). This effect has also been observed in studies on natural populations of intertidal foraminifera (Murray, 1983). That study documented a survivorship of individuals reaching the reproductive stage of ∼1.5% and a higher mortality in autumn in populations of *Haynesina depressula*. Individuals in Experiment 1 of this study included all size fractions (125–500 µm), with a higher amount of small individuals according to the natural population in the sample, and were sampled in autumn which might have effected mortality rates in this experiment. Summarizing, differences in mortality rates in laboratory experiments may occur as a result of different cultivation techniques, different distribution of ontogenetic stages within the experimental setup, the sampling season or the characteristics of the respective populations inhabiting the sampling area.

### Size-related carbon and nitrogen content, processing and intraspecific carbon and nitrogen scaling

Foraminifera play an important role in intertidal sediment biogeochemical fluxes, not only because of their high abundance, but also because of their role as primary consumers and detritivores. Foraminiferal nutrient cycling is also controlled by the abundances of intraspecific size classes and their relative contributions to the carbon and nitrogen pool. Larger *A. tepida* specimens contained considerably higher amounts of TOC and TN and showed higher pC and pN intake per individual (Table 1). Individual volume-specific carbon content typically shows an exponential relationship, which was also observed experimentally in several marine protists (e.g. Menden-Deuer and Lessard, 2000). Here, the results show values for pooled individuals within a size range, which should help to estimate contributions of foraminiferal populations to TOC and TN pools in intertidal environments (Fig. 3). Individual pC and pN intake of *A. tepida* was high and comparable with that of abundant foraminiferal species in productive shelf environments (compare with Enge et al., 2014, 2016). Also, contributions of total populations of *A. tepida* to phytodetritus processing was within the range found for foraminiferal key species in shelf environments (e.g. Uvigerinids, Enge et al., 2014, 2016). In studies at the northern Wadden Sea (Beukema, 1976; Reise et al., 1994), the biomass of macrofauna in tidal flats has been estimated to reach maximum values of ∼50–65 g ash-free dry weight m^−2^ (∼25–33 g TOC m^−2^, estimated from Salonen et al., 1976), and the biomass of mesofauna to reach ∼1 g ash-free dry weight m^−2^ (∼500 mg TOC m^−2^). In comparison, the maximum biomass of living *A. tepida* on our sampling date in April 2015 was ∼500 mg C m^−2^ (within the 0–1 cm sediment layer). Therefore, the biomass of *A. tepida* can at times approach those of maximum values of mesofauna, confirming a major contribution of *A. tepida* to the intertidal C and N pool at times of high abundances. Analogous, oxygen respiration measurements on intertidal foraminifera, including *A. tepida*, reported a high contribution of aerobic remineralization in intertidal mudflats (Cesbron et al., 2016).

On the sampling day at the end of April 2015, there were high abundances of *A. tepida* in the samples at Station 1 and Station 2, close to the shoreline. The station behind the tideway, Station 3, showed much lower numbers of *A. tepida*, most likely because of stronger exposure to currents at this sampling spot. The remarkably high abundances of living *A. tepida* individuals in the 125–250 µm size fraction indicates the recent occurrence of a reproductive event close to the sampling date. The size-specific content of and increased specific demand for food in smaller individuals are shown in the results of Experiment 2. This can be explained by the need to invest in growth and structure. The scaling relationships of weight-specific nitrogen content (Fig. 4A, Nitrogen) and demand for phytodetrital nitrogen (Fig. 4B,C, Nitrogen), show a prominent similarity between weight-specific TN content and demand for phytodetrital nitrogen per cytoplasmic nitrogen content (b ∼0.75, in all cases). This is possibly related to an orientation of food demand on the limiting nutrient nitrogen. The high cytoplasmic ratios of pN:TN in contrast to lower pC:TOC ratios in *A. tepida* (Fig. 2) also demonstrate a high nitrogen demand. A size-related metabolic scaling relationship of 0.75 for interspecific respiration is also present among several foraminiferal species (Geslin et al., 2011). This is consistent with theories explaining relationships between metabolic activities and organismic mass, surface area, or size of structural elements (Brown et al., 2004; Kleiber, 1932; Kooijman, 2010). The scaling of phytodetrital C or N intake per individual weight or per cytoplasmic TOC or TN shows a much higher relative intake and incorporation of phytodetritus at smaller size classes (Fig. 4B,C). This might indicate a higher turnover of C and respiratory losses in larger individuals and a stronger C retention in smaller ones, due to an increased incorporation into cytoplasmic components. Additionally, the high pC:TOC and especially high pN:TN in the young individuals of Experiment 1 (Fig. 2) underlines a strong demand for food-derived nitrogen sources in young individuals.

The extrapolation of cytoplasmic TOC, TN, pC and pN in *A. tepida* to the core material demonstrates the strong effect of high abundances of small individuals on the contributions to carbon and nitrogen pools and phytodetritus processing in populations of *A. tepida* (Fig. 3, Table 1). The relative amounts of TOC, TN, pC and pN at Station 1 and Station 2 are dominated by the 125–250 µm fraction (Fig. 5), expressing a strong effect of the size-specific scaling of phytodetritus intake (Fig. 4) at times after reproduction events.

The high total biomass of *A. tepida* (e.g. 136 ind cm^−3^ at Station 2), the fact that the highest concentrations of *A. tepida* are usually found within the uppermost layer of 0–0.5 cm (Cesbron et al., 2016; Thibault de Chanvalon et al., 2015) or 0–0.25 cm sediment (Alve and Murray, 2001) and the ability of foraminifera to expand their cytoplasm far beyond their test size, can reduce the space available per individual. Examples for pseudopodial expansion in the genus *Ammonia* are shown in Debenay et al. (1998). This cytoplasmatic expansion possibly increases the impact of population-density depending factors (e.g. space, food resources) and may result in intraspecific competition within *A. tepida* populations at times of reproduction and shortly thereafter. This might be a critical factor controlling the cyclicity observed in population densities of intertidal foraminifera. A flexible response to food supply as observed in Experiment 1 in adult individuals might therefore help to withstand such competitive pressures at times of resource shortage. However, further investigations are necessary to support this hypothesis.

Given the production of particulate carbon in the sampling area (e.g. Tillmann et al., 2000), the pC values and abundances of *A. tepida* derived from this study, the *A. tepida* community in the sampling area is capable of processing of a maximum of ∼11% of phytodetrital TOC in the productive season. With the corresponding nitrogen values from the experiment, the contribution of *A. tepida* to the processing of particulate organic nitrogen derived from phytodetritus can be estimated to max. ∼6%. There is not much known about foraminiferal nitrogen regeneration from organic matter, but isotope tracer studies in the Arabian Sea have shown a high contribution to the processing of phytodetrital nitrogen by benthic foraminifera (Enge et al., 2016). Feeding experiments with *A. tepida* have also shown a coupling of carbon and nitrogen intake from *D. tertiolecta* detritus (Wukovits et al., 2017), which is also apparent in the present study (see Fig. S2). The patterns of cytoplasmic nitrogen content in this study are similar to the carbon patterns (Fig. 1). Food-derived nitrogen in *A. beccarii* is most probably used to synthesize amino acids as an element of the OM in the foraminiferal test (Nomaki et al., 2015). The increased production of such amino acids in the small size fraction of this experiment might therefore explain the higher demand for nitrogen in the younger specimens (Fig. 4B,C, Nitrogen). Further, *Ammonia sp.* reportedly stores assimilated nitrate under anoxic and dysoxic conditions (Nomaki et al., 2014, 2016), although this was most likely not the case in this study (oxic conditions). An increase in cytoplasmatic nitrogen pools or nitrogen storage and might have resulted in a decoupling of pC and pN under the different treatments (compare with Fig. S2).

The switch of the food sources from the period prior to the experiment (fed regularly with living algae) to phytodetritus at the experiments, might have affected the carbon and nitrogen processing in *A. tepida*, probably by lowering the intake to adapt to the new food source. However, natural switches of food availability occur in estuaries due to seasonal changes in algal community structures, changes in transportation rates of riverine OM, or increased availability of dead algae matter following a phytoplankton or microphytobenthos bloom. Generally, there is a broad range of potential algal food sources in the natural environment of the German Wadden Sea (Scholz and Liebezeit, 2012a,b; Tillmann et al., 2000). The foraminiferal specimens in this study were fed with monospecific cultures of living *D. tertiolecta* for maintenance and the experiments were carried out with phytodetritus of this algae (to track the isotope signal of the food source). These diets do not correspond with the natural variety of food sources for *A. tepida* in the German Wadden Sea. However, *A. tepida* individuals kept in the laboratory have previously shown a good response to *Dunaliella*-derived diets (Bradshaw, 1961; Linshy et al., 2014; Wukovits et al., 2017). Considering the abundance of several sources of OM as potential food with different nutritional value and processing rates in nature, results of this experiment might reflect a good estimate of the mean carbon and nitrogen processing of *A. tepida*.

In summary, adult individuals of *A. tepida* showed flexible patterns of phytodetritus processing as a response to different feeding modes. This could be interpreted as metabolic adaptation to different modes of food availability and represent a generalistic survival strategy in environments with strongly fluctuating food availability throughout the year. The overall content of TOC, TN, and phytodetrital intake (pC and pN) in *A. tepida* were high and comparable with foraminiferal key species in other productive ocean regions, reaching biomass values approaching that of maximum values of intertidal mesofauna. This supports the consideration of *A. tepida* as an important player in intertidal nutrient fluxes in seasons with high *A. tepida* abundances. Large individuals showed substantially higher contents of TOC and TN, and of phytodetrital intake. However, phytodetritus processing (specifically N) relative to individual size (dry mass) increased with decreasing individual size, most likely due to a higher energy demand to cover growth costs in juveniles. This higher relative food demand in young individuals affects the contributions to the total foraminiferal TOC and TN pools and phytodetritus uptake, increasing the impact of the small size fraction on intertidal nutrient cycling.

## MATERIALS AND METHODS

### Sampling site and preparations for feeding experiments

**Fig. 6.**
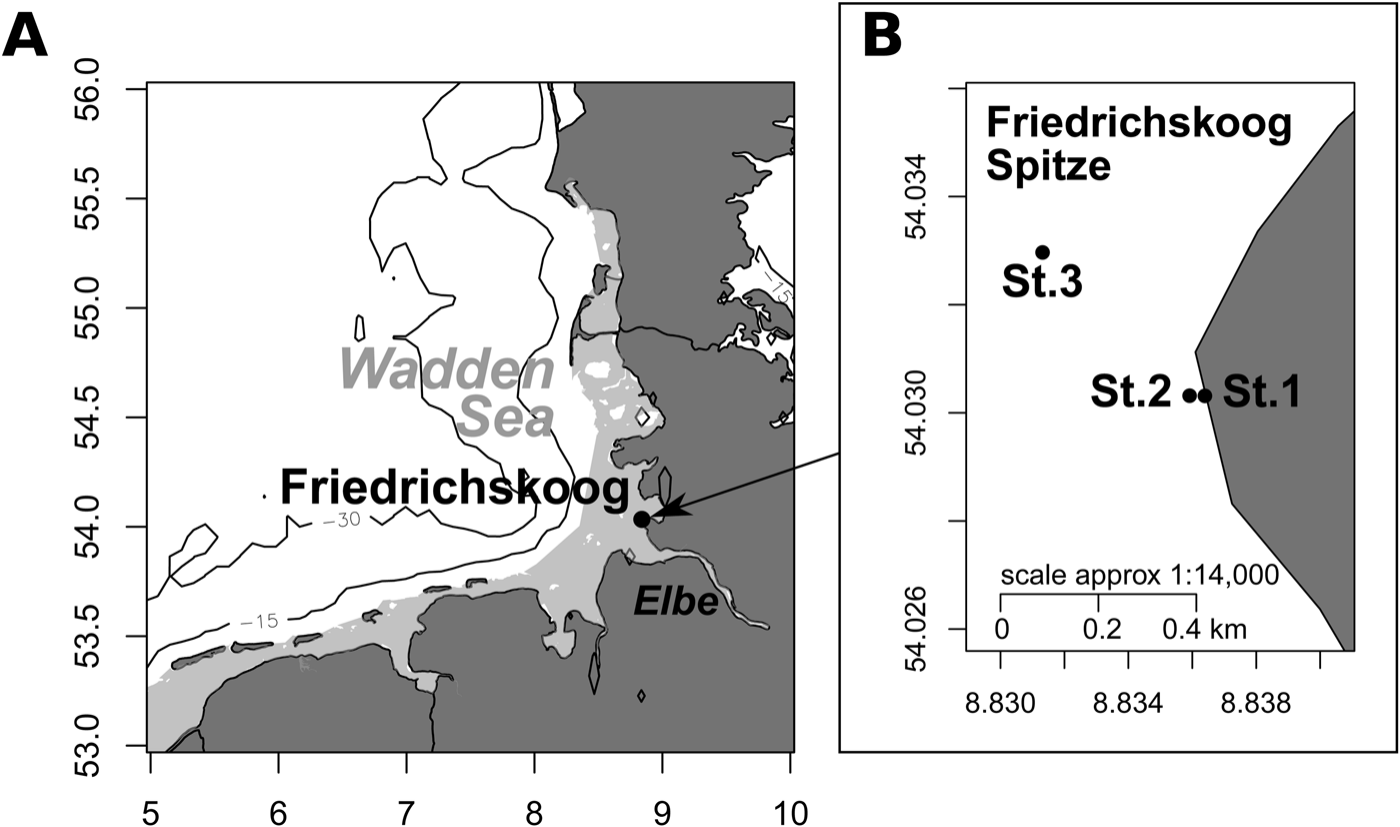
**Sampling area.** (A) Location of the intertidal mudflats at Friedrichskoog/German Wadden Sea. (B) Position of sampling spots for sediment core samples (St. 1=Station 1, St. 2=Station 2, St. 3=Station 3. One sediment core was taken at each position).

Foraminifera were collected at low tide in the intertidal mudflat of the German Wadden Sea near Friedrichskoog, at the northern part of the Elbe Estuary (Fig. 6). Samples were taken in April and August 2015. For the feeding experiments, the sediment surface was sampled to collect high numbers of living individuals of *A. tepida*. The sediment was pre-sieved at the sampling sites through 500 µm and 125 µm mesh to remove larger meiofauna and organic particles. At the University of Vienna, living *A. tepida* individuals were picked and collected in crystallizing dishes containing a thin layer of Wadden Sea sediment (particle size <60 µm). Traces of movement on this sediment and pseudopodial activity served as vital signs, as well as protoplasmic color (rich yellow) and particle accumulation around the aperture (Moodley et al., 2000; Nomaki et al., 2005, 2006). Specimens were repeatedly (every 4–5 days) fed with living *Dunaliella tertiolecta* (Chlorophyta) until the start of the experiments and the green coloration of foraminiferal cytoplasm served as another indicator of vital activity. Additionally in April, three sediment cores were taken (4.5 cm diameter) on the vertices of an acute-angled triangle (Station 1 and Station 2 were ∼120 m off the shore line, ∼65 m apart and covered with diatom mats, Station 3 was located behind a tide way ∼550 m off the shoreline). The uppermost cm of the sediment cores was stained with Rose Bengal and Ethanol to determine the abundances of living *A. tepida* individuals of the size fractions 125–250 µm, 250–355 µm and >355 µm.

To produce an isotope-labeled food source, a culture of *D.* *tertiolecta* was grown in f/2 medium (Guillard, 1975; Guillard and Ryther, 1962) enriched with 98 atom % ^13^C (NaH^13^CO_3_, Sigma-Aldrich) and 98 atom % ^15^N (Na^15^NO_3_, Sigma-Aldrich). The culture was harvested by centrifugation, rinsed three times in sterile, carbon and nitrogen free artificial seawater, frozen in liquid nitrogen and freeze-dried to produce artificial phytodetritus (Hunter et al., 2013; Wukovits et al., 2017).

### Sample preparation and analysis

After termination of the respective experiments (for a detailed description see sections ‘Experiment 1: Comparison of food uptake from a single high feeding pulse versus lower constant feeding’ to ‘Experiment 2: Size specific carbon and nitrogen processing’), foraminifera were removed from the experimental dishes and frozen at −20°C to stop metabolic activity. Specimens were carefully cleaned to remove adhering particles, transferred to tin capsules and dried overnight at 50°C. The tests were decalcified with (10–15 µl) 4% HCl. The remaining cytoplasm was dried in a final drying step for 3 days at 50°C. The emerging mass of CaCl_2_ through decalcification was subtracted from the dry weight by using the reaction equilibrium for CaCO_3_ and HCl. All glassware used for preparation was combusted at 500°C for 5 h, and picking tools and tin capsules were cleaned in a solution of dichloromethane and methanol (CH_2_Cl_2_:CH_4_O; 1:1, *v:v*) to remove organic contaminants.

Foraminiferal samples were analyzed at the Stable Isotope Laboratory at the University of Vienna for Environmental Research (SILVER). Cytoplasmic content of organic carbon or nitrogen and ratios of ^13^C/^12^C and ^14^N/^15^N were determined with an Isotope Ratio Mass Spectrometer (DeltaPLUS, Thermo Finnigan, Vienna, Austria) interfaced (ConFlo III, Thermo Finnigan) to an elemental analyzer (EA 1110, CE Instruments, Vienna, Austria). Atom% of the samples were derived from isotope ratio data and were calculated using the Vienna Pee Dee Belemnite standard (*R*) for C (*R*_VPDB_=0.0112372) and atmospheric nitrogen for N (*R*_atmN_=0.0036765), where *X* is ^13^C or ^15^N:
(2)
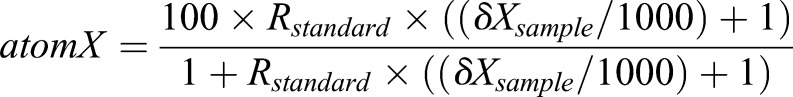


The excess (E) of heavy stable isotopes in the labelled samples was calculated to determine the individual content of phytodetrital carbon (pC) and nitrogen (pN) within foraminiferal cytoplasm (Middelburg et al., 2000) where *X* is ^13^C or ^15^N:
(3)
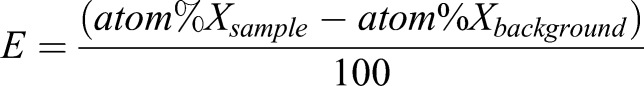


The product of isotope excess and total foraminiferal carbon and nitrogen content was used to calculate the amount of incorporated isotope *I*_iso_, and following the amount of phytodetrital carbon (pC) and nitrogen (pN) within the foraminiferal cytoplasm *I*_phyto_ (Hunter et al., 2013):
(4)
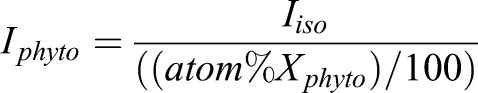


Statistics were calculated using R version 3.3.2 (corresponding packages for analysis of experimental data are listed in the respective sections below), and graphs were done using ggplot2.

### Experiment 1: Comparison of food uptake from a single high feeding pulse versus lower constant feeding

Foraminifera from the August 2015 batch were transferred to vials (d=4.5 cm, h=6 cm, 55–60 specimen per vial) containing 0.8 g heat sterilized Wadden Sea sediment (<63 µm) and covered with plankton net squares. The experiment started 2 weeks after sediment collection. To obtain triplicate samples for 2, 4, 7, 14, 21, and 28 day incubations for the three different culture conditions, 45 vials were prepared and placed in aquaria. Incubations conditions were as follows: constant feed 1=constant feeding at temperature cycles of 20°C:18°C; constant feed 2=25°C:23°C, single pulse=single high feeding pulse at 20°C:18°C=single pulse, each cycle was 12 h:12 h (Table 2); cycles were chosen to mimic natural diurnal temperature fluctuations; standard temperature fluctuations of incubation chambers: ±0.6 at 20°C. Each of the three setups was incubated within one aquarium filled with filtered North Sea Water and aerated with ambient air, to support the stability of culture conditions during the experimental period. At the beginning of the experiment, labelled phytodetritus (*D. tertiolecta*) was introduced into the respective vials at day 0 for the single pulse approach (∼1670 mg C m^−2^) and the constant feeding approach (∼560 mg C m^−2^). In constant feeding mode, additional food (∼560 mg C m^−2^) was supplied every third day (Table 2). The amount of added carbon was adapted according to planktonic primary production (daily gross particulate production approximately 500–2000 mg C m^−2^ day^−1^) in the German Bight (Tillmann et al., 2000) during summer months. Oxygen, pH and salinity were recorded on a regular basis during the run of the experiment to retain environmental conditions within the optimum range. Some replicates contained dead individuals at the time of sampling. Dead individuals were excluded from further analysis. Towards the end of the experimental period, some replicates had to be pooled to obtain sufficient amounts of live specimens for further elemental and isotopic analysis.

**Table 2.**
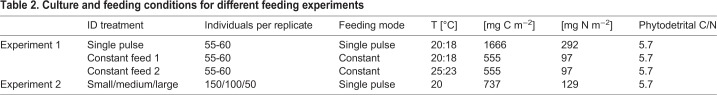
**Culture and feeding conditions for different feeding experiments**

### Experiment 2: Size-specific carbon and nitrogen processing

Sediment samples collected in April 2015 were sieved to obtain three size classes (small: 125-250 µm; medium: 250-355 µm; large: >355 µm). Foraminifera were picked out and triplicate samples for each size class (50–150 individuals, depending on size class) were set up in crystallizing dishes, containing 280 ml artificial Seawater (modified after Gattuso et al., 1998) and incubated at 20°C, starting four weeks after field sampling. Samples were taken 2 and 4 days after the initial feeding pulse of *D. tertiolecta* phytodetritus (Table 2) and processed for EA-IRMS analysis as described above.

Total organic carbon (TOC), total nitrogen (TN), and pC and pN of foraminiferal cytoplasm were compared between size classes and between sampling days (Mann–Whitney *U*-test), to determine the effect of incubation time and size class on cellular element content or phytodetritus intake. Then, the data were log normalized to fit linear regressions (general least squares, GLS) to explore the relationships of size-related intraspecific variables (individual dry weight, TOC, TN) and phytodetritus intake (pC, pN). The back-transformed regression equations were used to interpret the proportionality of size-dependent TOC and TN content and individual food intake, where, e.g. an isometric relationship (slope of the regression, b ∼1) describes an even rise of the measured variables with foraminiferal size (no difference in size-related cytoplasmic TOC and TN or phytodetritus intake), and a lower slope (b<1) describes a negative proportionality, i.e. a decrease of cytoplasmic TOC and TN levels and phytodetritus intake with increasing size.

Finally, foraminiferal TOC, TN, pC and pN were projected on the three size fractions in a natural population of *A. tepida* by using the abundance data from the Rose Bengal stained sediment cores, to estimate the effect of size-dependent phytodetritus intake based on the natural size distribution of an *A. tepida* community and to assess the total contribution of this community to the sediment bulk TOC and TN pool.

Data analysis was done using R and related packages ggplot2, dplyr, doBy (Højsgaard and Halekoh, 2016; R Development Core Team, 2008; RStudio Team, 2015; Wickham, 2009; Wickham et al., 2017).

## Supplementary Material

Supplementary information
