# Supplementary material for: Food supply and size class depending variations in phytodetritus intake in the benthic foraminifer Ammonia tepida
Source: Biol Open. 2018 Mar 14;7(4):bio030056. doi: 10.1242/bio.030056 (PMC5936058; doi:10.1242/bio.030056)
Supplement: Supplementary information [file biolopen-7-030056-s1.pdf]

Table S1: Data of Elemental Analysis and Isotope Ratio Mass Spectrometry for analyzed individuals of *A. tepida*.

	Sample	Rep.	Nr Ind. analysed	sample weight [mg]	δ 15N/14N	AT% 15N/14N	δ 13C/12C	AT% 13C/12C	µg N	µg C
Exp. 1	SP 2d 1	1	39	0.938	8360.9	3.329	285.6	1.417	6.14	29.98
	SP 2d 2	2	37	0.856	8638.9	3.424	312.7	1.446	4.83	22.76
	SP 2d 3	3	42	0.902	10202.2	3.957	351.7	1.489	5.64	29.52
	SP 4d 1	1	43	1.186	7089.7	2.890	235.2	1.362	7.59	38.61
	SP 4d 2	2	38	0.973	5357.2	2.285	173.1	1.295	5.64	30.52
	SP 4d 3	3	33	0.983	7941.9	3.184	286.3	1.418	5.66	28.42
	SP 7d	1	21	0.859	8710.0	3.448	333.4	1.469	4.25	18.10
	SP 14d 1	1	25	0.986	3808.6	1.738	133.7	1.252	4.69	16.87
	SP 14d 2	2	30	1.018	8466.8	3.365	276.1	1.407	5.21	22.70
	SP 14d 3	3	18	0.940	7780.6	3.129	235.1	1.362	4.19	17.31
	SP 28 d	1	23	0.790	10702.1	4.127	398.2	1.539	3.13	12.60
	SP 28 d	2	28	0.920	8112.2	3.243	322.3	1.457	3.54	12.94
	SP 28 d	3	21	0.891	9125.0	3.590	326.4	1.461	3.42	14.23
	CFnorm 2d	1	35	0.986	9094.0	3.580	360.4	1.498	5.51	24.15
	CFnorm 2d	2	40	0.874	10150.5	3.940	374.5	1.513	5.64	26.10
	CFnorm 2d	3	31	1.008	9690.5	3.783	399.8	1.541	4.64	19.51
	CFnorm 4d	1	30	n.a.	10198.4	3.956	353.1	1.490	4.43	21.27
	CFnorm 4d	2	34	0.973	9839.7	3.834	362.4	1.500	4.79	22.20
	CFnorm 4d	3	26	0.871	9181.8	3.610	339.3	1.475	3.80	17.13
	CFnorm 7d	1	21	0.803	9338.5	3.663	365.2	1.503	3.04	12.71
	CFnorm 7d	2	26	0.862	10791.4	4.157	415.0	1.557	3.40	14.48
	CFnorm 7d	3	22	0.781	9840.8	3.835	361.1	1.499	3.06	13.47
	CFnorm 14 d	1	47	1.130	12580.3	4.757	459.5	1.606	5.83	26.22
	CFnorm 28d young	1	35	0.487	25716.5	8.948	891.5	2.071	1.83	7.61
	CFnorm 28 d adult	1	24	0.974	13922.2	5.203	561.9	1.716	3.65	15.00
	CFwarm 2d 1	1	25	0.801	8228.7	3.283	313.8	1.448	2.79	12.36
	CFwarm 2d 2	2	25	0.904	7132.7	2.904	272.9	1.403	3.78	16.72
	CFwarm 2d 3	3	37	1.029	11463.4	4.383	424.3	1.567	4.51	20.60
	CFwarm 4d 1	1	29	1.058	10860.9	4.180	348.4	1.485	4.19	20.64
	CFwarm 4d 2	2	36	1.115	14522.7	5.401	490.6	1.639	6.02	28.93

Biology Open • Supplementary information

	CFwarm 4d 3	3	18	0.828	15950.4	5.869	539.6	1.692	3.02	14.24
	CFwarm 7d 1	1	22	0.871	9798.9	3.820	383.1	1.523	2.75	11.20
	CFwarm 7d 2	2	20	0.856	10559.3	4.078	407.5	1.549	3.20	13.28
	CFwarm 7d 3	3	31	0.978	11861.8	4.517	507.9	1.658	4.91	19.37
	CFwarm 14d alle	1	31	1.087	8956.6	3.533	388.9	1.529	3.92	14.85
	CFwarm 28d alle	1	16	0.888	5394.7	2.298	190.9	1.314	3.65	13.48
Exp. 2	125 - 250 µm 2d 1	1	146	1.583	4119.6	1.848	850.0	2.026	7.50	36.40
	125 - 250 µm 2d 2	2	112	1.870	4132.4	1.853	967.7	2.153	6.30	29.07
	125 - 250 µm 2d 3	3	157	1.697	6230.1	2.590	1286.5	2.493	9.10	48.01
	250 - 355 µm 2d 1	1	99	2.807	3228.3	1.531	487.7	1.636	15.29	116.82
	250 - 355 µm 2d 2	2	94	2.765	4518.6	1.989	601.8	1.759	15.58	120.70
	250 - 355 µm 2d 3	3	100	2.899	2011.0	1.095	283.8	1.415	15.92	120.89
	> 355 µm 2d 1	1	55	2.793	3782.7	1.729	515.8	1.666	13.83	110.33
	> 355 µm 2d 2	2	61	2.930	3203.1	1.522	439.4	1.584	17.83	142.08
	> 355 µm 2d 3	3	54	2.764	5331.0	2.276	841.1	2.017	15.21	100.69
	125 - 250 µm 4d 1	1	134	1.828	8403.7	3.343	1740.1	2.972	10.46	49.85
	125 - 250 µm 4d 2	2	130	1.815	6762.9	2.776	1367.2	2.578	9.47	43.41
	125 - 250 µm 4d 3	3	133	2.220	6078.4	2.537	1243.4	2.447	9.41	42.34
	250 - 355 µm 4d 1	1	96	3.001	4698.9	2.053	901.2	2.081	15.35	76.42
	250 - 355 µm 4d 2	2	95	2.885	3422.4	1.601	564.1	1.719	13.17	58.80
	250 - 355 µm 4d 3	3	91	2.721	4635.2	2.031	878.1	2.057	13.23	66.91
	> 355 µm 4d 1	1	51	2.804	3446.2	1.609	540.7	1.693	14.46	77.29
	> 355 µm 4d 2	2	48	2.899	2958.7	1.435	449.5	1.595	14.83	73.97
	> 355 µm 4d 3	3	50	3.084	5730.0	2.416	1033.4	2.223	15.96	85.61

Initial setup: 3 replicates per day for each experiment, each containing 55 - 60 Individuals *A. tepida*. The number of realized replication in the analysis is indicated in the third column.

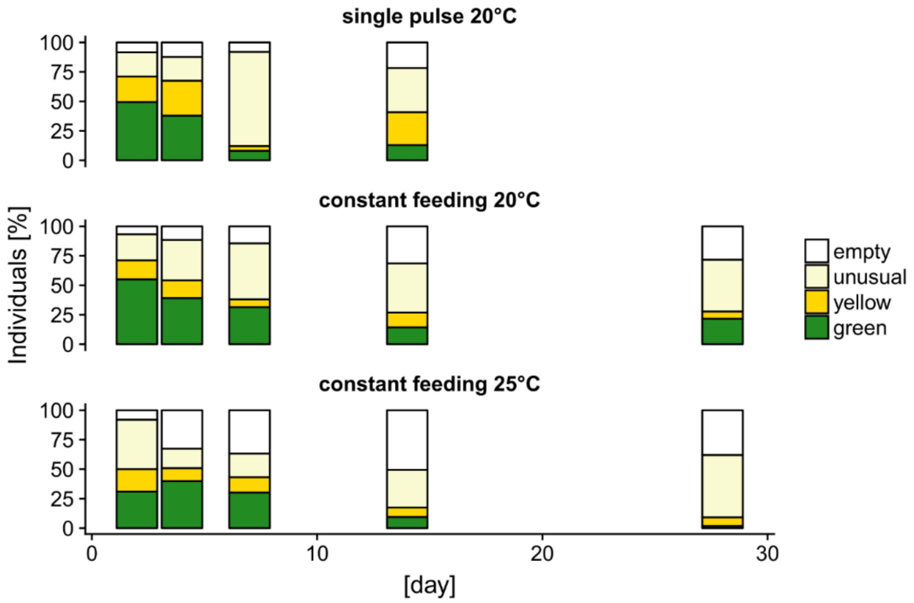

Fig. S1: Total percental amount of foraminiferal appearance according to their cytoplasmic coloration at the respective sampling day. Empty refers to transparent individuals, unusual cytoplasmic coloration includes individuals with unusually pale color or retreated cytoplasm, yellow individuals are characterized by a predominantly bright, yellow color and some inclusions of green indicating phytodetritus intake, green individuals show an extensive coloration according to the offered phytodetrital food source. Only yellow and green individuals were analyzed, empty and unusual colored individuals were considered to be dead or deceasing. The decrease of the survival in the progress of the experiment, indicates an effect of time on the survival of the Individuals.

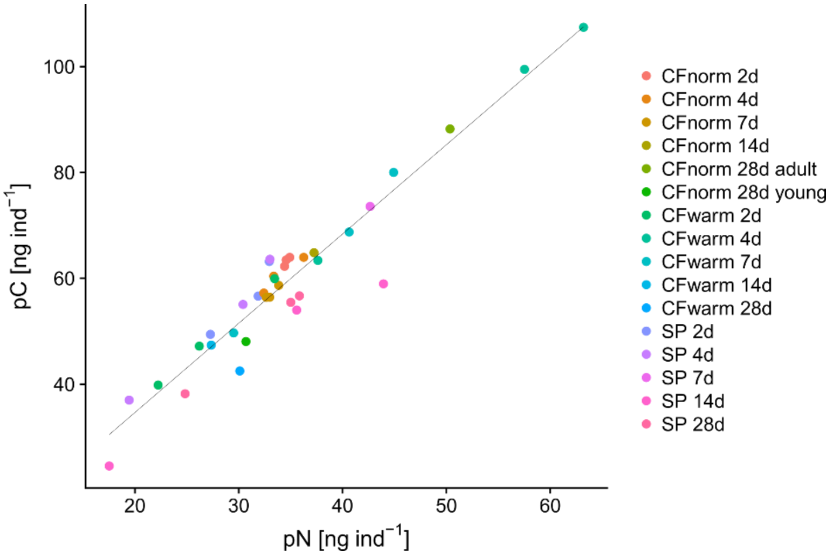

Fig. S2: Relationship of cytoplasmatic pN and pC in *A. tepida*. Colors are showing the different treatments, sampling days (CFnorm = constant feed 1, 20:18°C; CFwarm = constant feed 2, 25:23°C; SP = single pulse, 20:18°C; $R^2 = 0.92$, $p < 0.001$).

Biology Open • Supplementary information
